# Effects of dietary cecropin on growth performance, diarrhea rate and intestinal health of nursery Hainan pigs

**DOI:** 10.3389/fmicb.2024.1298703

**Published:** 2024-04-03

**Authors:** Kun Ouyang, Ting Chen, Ruiping Sun, Yali Xie, Qi Qi, Xiang Li, Jie Liu, Quanwei Liu, Limin Wei

**Affiliations:** ^1^Hainan Key Laboratory of Tropical Animal Breeding and Epidemic Research, Institute of Animal Husbandry and Veterinary Research, Hainan Academy of Agricultural Sciences, Haikou, China; ^2^College of Animal Science, South China Agricultural University, Guangzhou, China; ^3^Sanya Institute, Hainan Academy of Agricultural Sciences (Hainan Experimental Animal Research Center), Sanya, China

**Keywords:** nursery Hainan piglet, cecropin, growth performance, diarrhea rate, gut health

## Abstract

Antimicrobial peptides could inhibit the growth of harmful bacteria and promote the growth performance in weaned piglets. Here, we investigated the effects of dietary supplementation with cecropin antimicrobial peptides (CAP) on growth performance, diarrhea rate, intestinal health in nursery Hainan piglets. For this, 120 healthy nursery Hainan male piglets (13.29 ± 0.29 kg, 44 days old) were randomly divided into 5 groups—a control (CON) group (fed a basal diet), an antibiotic control (AC) group (fed a basal diet supplemented with 250 mg/kg colistin sulfate); and 3 experimental groups (provided the basal diet supplemented with 250, 500, or 1,000 mg/kg CAP). Pre-feeding lasted 7 days and the official period lasted 40 days. The results showed that compared with the CON group, dietary supplementation of 500 mg/kg CAP had significantly increased the average daily gain (ADG, *p* < 0.05), while the feed conversion ratio (FCR) and diarrhea rate were markedly reduced (*p* < 0.05), serum total protein (TP), albumin, IgA, IgM, and globulin concentrations were significantly increased (*p* < 0.05), where serum aspartate aminotransferase (AST) level was significantly reduced (*p* < 0.05), and it also increased the villus height and the villus height-to-crypt depth ratio in the jejunum, reduced the serum *D*-lactic acid concentrations and diamine oxidase activity, and increased the expression level of ZO-1 and occludin in the jejunum and ileum (*p* < 0.05), the relative abundance of Firmicutes, *Lactobacillus*, and *Limoslactobacillus* in the colon were increased (*p* < 0.05), whereas that of *Streptococcus* and *Escherichia*–*Shigella* were reduced (*p* < 0.05). These results indicated that dietary supplementation with 500 mg/kg CAP could improve the growth performance, reduce the diarrhea rate, improve the serum immunity, intestinal health of nursery pigs.

## Introduction

Weaning is an important period in modern intensive pig farming (Sun et al., 2022); however, the digestive and immune systems of piglets are immature at this stage. Studies have shown that the activity of digestive enzymes such as pepsin, amylase, chymotrypsin, and trypsin in piglets were decreased significantly within 1 week of weaning, which creates problems for their digestion (Jensen et al., 1997). Meanwhile, the process of weaning can induce stress in piglets, leading to various adverse effects such as diarrhea, reduced growth rate, disruption of intestinal flora, and potentially fatal outcomes (Su et al., 2022). These consequences impose a substantial economic burden on breeding enterprises (Liu et al., 2022). Bacterial infections, and in particular those associated with enterotoxigenic *Escherichia coli*, are the main causes of diarrhea in piglets (Xu et al., 2020). Hainan pigs, a native pig breed in Hainan province, China, are widely recognized for their superior meat quality, robust disease resistance, and remarkable fertility. Nonetheless, these pigs do exhibit certain drawbacks, including low weight and a heightened prevalence of diarrhea among weaned piglets (Xiao, 2011). In order to improve or reduce the impact of feed and environmental changes after weaning, and improve the growth performance and economic benefits of piglets, enterprises usually use antibiotics such as colistin sulfuric, chlortetracycline, oxytetracycline etc. to reduce weaning stress of piglets (Drider et al., 2022). Colistin sulfuric was used to treat bacterial infections such as *Escherichia coli* and *Salmonella*, and it was the preferred drug for treating intestinal infections in the past (Kwa et al., 2007), therefore, it was often used as a feed additive in piglet breeding to improve intestinal health and reduce the diarrhea rate of piglets. However, some bacterial families, such as Enterobacteriaceae, are resistant to nearly all antibiotics (Gresse et al., 2017), and antibiotics frequently cause dysbiosis and reduce microbiota stability by limiting the growth and proliferation of both pathogenic and nonpathogenic bacteria (Salim et al., 2018). Moreover, the overuse of antibiotics has consequences for the environment as well as for human health, and the use of antibiotics in diets has been banned in China since July 1, 2020. Accordingly, there is an urgent need to identify and develop safe and efficient alternatives to antibiotics.

Antimicrobial peptides (AMPs) comprise a class of small peptides widely present in animals and plants. They have little to no toxicity and have been reported to possess antibacterial (Vanzolini et al., 2022), antiviral (Huang et al., 2013), antitumor (Jaber et al., 2021), and immune regulatory effects (Pane et al., 2016). Additionally, unlike antibiotics, AMPs exert bacteriostatic effects by binding to bacteria and destroying their cell membranes, rendering it difficult for bacteria to develop resistance to these peptides (Benke et al., 2017). Cecropins are AMPs derived from insects, including the domestic silk moth *Bombyx mori*, and are key components of their innate immune systems (Nesa et al., 2019), and at present, cecropin antimicrobial peptides had been found to be divided into six types: A, B, C, D, E, and P1. They are currently the subject of intense research interest because of their broad-spectrum antibacterial activity and difficulty in generating drug resistance. Dietary supplementation with cecropin A can alleviates inflammatory bowel disease (IBD) in C57BL/6 mice through decreasing harmful gut microflora and specifically enhancing beneficial gut microflora (Zhai et al., 2019). The supplementation of cecropin AD in the diet can alleviate piglet diarrhea caused by enterotoxin-producing *E. coli* (Wu et al., 2012). Additionally, study found that cecropin A supplementation can increase the protein expression of ZO-1, claudin-1, and occludin, as well as enhance the barrier function of porcine intestinal epithelial cells by inhibiting the MEK/ERK pathway (Zhai et al., 2018). Research has shown that cecropin B can disrupt the anionic cell membranes of Gram-negative bacteria, thus producing bacteriostatic effect on them (Sallum and Chen, 2008). *In vivo* experiment, cecropin B was injected into dairy goat mammary gland which inhibited the mastitis caused by *Staphylococcus aureus* (Luo et al., 2013). And cecropins also has antiviral effects, studies showed that cecropin D and cecropin P1 can inhibit porcine reproductive and respiratory syndrome virus infection and reduce cell apoptosis (Guo et al., 2014; Liu et al., 2015). However, the functions of C and E have not been reported yet. Over recent years, the role of cecropins in the treatment of intestinal inflammation and the regulation of intestinal flora has gradually attracted attention. As a substitute for antibiotics, cecropin has unique research significance and application prospects, but in the research of cecropins on weaned piglets, how to alleviate weaning stress in piglets and optimum addition amount is still unclear and there are few studies about the impact on intestinal flora structure, especially in Chinese local pig breeds.

In this study, we supplemented the diets of nursery Hainan piglets with cecropin antimicrobial peptides (CAP) or the colistin sulfate and compared their effects on growth performance, immune status, intestinal morphology, intestinal tight junction and intestinal microorganismal structure. Our findings help to shed light on the different mechanisms for the actions of cecropins or antibiotics, and provide reference basis for the application of cecropins in the actual breeding of piglets.

## Materials and methods

### Animal ethics

All the experimental procedures applied in this study were reviewed and approved by the Experimental Animal Ethics Committee of Animal Husbandry and Veterinary Research Institute, Hainan Academy of Agricultural Sciences.

### Materials

Silkworm cecropin A was purchased from Wuhan Woxuan Biotechnology Co., Ltd. (Wuhan, China). The purity of the peptides was 98% as determined by high-performance liquid chromatography.

### Animal treatment

A total of 120 healthy Hainan nursery male piglets (initial body weight: 13.29 ± 0.29 kg, 44 days old) were allotted into five treatments based on body weight, in a completely randomized block design (4 replicates per group, 6 piglets per replicate): a control (CON) group (fed a basal diet), an antibiotic control (AC) group (fed with the basal diet supplemented with 250 mg/kg colistin sulfate); and 3 experimental groups (fed with the basal diet supplemented with 250, 500, or 1,000 mg/kg CAP, respectively). The experimental diets were formulated to meet Chinese “*Standards for Pig Feeding*” (NY/T 65-2004) (NY/T 65-2004, 2004). The composition and nutritional level of the basal diet are shown in Table 1. The piglets were fed 3 times one day in an open barn with *ad libitum* access to food and water. The pre-feeding period lasted for 7 days and the official period for 40 days. All experiments were approved by the Animal Care Committee of South China Agricultural University, and immunization was implemented following the breeding management regulations of the Yongfa Experimental Base of the Animal Husbandry and Veterinary Research Institute of the Hainan Academy of Agricultural Sciences. From the beginning (day 1) to the end (day 40) of the experimental period, all the piglets were weighed at the 40 days and the 40 days feed intake for each group was calculated; the diarrhea status was recorded every day. The average daily feed intake (ADFI), average daily weight gain (ADG), and feed conversion ratio (FCR) were also calculated. The occurrence of diarrhea for each piglet was visually assessed each afternoon according to the method of Hart and Dobb (1988). Scores were 0 = normal, firm feces; 1 = possible slight diarrhea; 2 = definitely unformed, moderately fluid feces; or 3 = very watery and frothy diarrhea, The occurrence of diarrhea was defined as maintaining fecal scores of 2 or 3 (Montagne et al., 2004). The fecal score of piglets of each litter was recorded daily, their diarrhea rate during days 1–40 was also be calculated, and the diarrhea rate (DR, %) was calculated by the number of diarrhea cases/(total number of pigs × trial days).

**Table 1:** Composition and nutrient levels of the basal diet.

Ingredient	Content	Nutrient levels^2^	
Corn	68.20	DE, (MJ/kg)	13.31
Soybean meal	22.20	EE	5.80
Fishmeal	2.50	CP	19.30
Soybean oil	3.00	CF	0.26
Lys	0.10	Lys	1.20
Premix^1^	4.00	Met + Cys	0.54
		Ca	0.64
Total	100.00	P	0.46

^1^Premix was provided per kg of ration. Vitamin (V) A, 3,000 IU; VD_3_, 440 IU; VE, 26.48 IU; VK_3_, 0.5 mg; VB_2_, 11 mg; VB_6_, 2.2 mg; nicotinamide, 33 mg; *D*-calcium pantothenate, 2 mg; Se (as sodium selenite), 0.1 mg; Cu (as copper sulfate), 40 mg; Fe (as ferrous sulfate), 60 mg; Mn (as manganese sulfate), 2 mg; Zn (as zinc sulfate), 48 mg; I (as calcium iodide), 0.24 mg. ^2^Ether extract (EE), crude protein (CP), crude fiber (CF), calcium (Ca), and phosphorus (P) were measured values; the rest were calculated values.

### Sample collection

After the test, we randomly select 6 piglets with body weight close to average for euthanasia in each group. Blood (5 mL) was collected from the anterior vena cava into a coagulation tube and stored at 4°C. The gut was taken through a sterile laparotomy, then 1 cm of the middle parts of the jejunum and ileum were collected and placed in neutral formalin for histological analysis. The mucosa of jejunum and ileum was collected in centrifuge tubes and then immediately placed in liquid nitrogen and stored at −80°C for analysis of mRNA expression. Digesta from the colon were obtained using centrifuge tubes, immediately placed in liquid nitrogen, then stored at −80°C for analysis of the microbial community structure.

### Detection of serum indicators

After centrifugation at 3,000 × *g* at 4°C for 10 min, the serum (supernatant) was collected and stored at −80°C until used for the detection of serum physiological and biochemical indicators, including total protein (TP), albumin (ALB), globulin (GLB), IgA, IgM, IgG, alanine aminotransferase (ALT), and aspartate aminotransferase (AST). IgA (H108-1-2), IgG (H106-1-2), IgM (H109-1-2), *D*-lactate acid (H263-1-2), and diamine oxidase (DAO, A008-1-1) contents were measured by ELISA on a Spectra Max iD5 multifunctional microplate reader while serum TP, ALB, GLB, ALT and AST levels were determined using an automatic biochemical analyzer (LABOSPECT008A, HITACHI, Japan). ELISA kits were purchased from Nanjing Jiancheng Bioengineering Institute (Nanjing, China). The samples were measured by following the manufacturers’ instructions.

### Measure pH of the stomach and small intestine

We took the contents of the anterior, middle, and posterior segments of the stomach, duodenum, jejunum, and ileum separately, diluted them tenfold with deionized water, stirred for 5 min, and calculated the pH value using a pH meter (testo 205, Germany).

### Histological analysis

Tissue samples from the middle jejunum and ileum were fixed in 4% paraformaldehyde, paraffin-embedded, cut into 5 μm-thick sections, stained with hematoxylin and eosin (H&E), and imaged using a panoramic slide scanner (PANNORAMIC, 3DHISTECH, Budapest, Hungary). Villus length, crypt depth, and the villus-height-to-crypt-depth (V/C) ratio were measured using ImagePro Plus 6.0 (Media Cybernetics, Maryland, United States).

### RNA isolation and quantitative real-time PCR

Small pieces of jejunal and ileal mucosa were homogenized in liquid nitrogen. Total RNA was isolated from 30 to 50 mg of homogenized mucosal tissue using a RNeasy Mini Kit (Qiagen, Germany). Extracted RNA was reverse transcribed into cDNA using FastKing gDNA Dispelling RT SuperMix (TIANGEN, Beijing) according to the manufacturer’s instructions. Quantitative, real-time PCR was performed using QuantiNova SYBR Green PCR Master Mix (Qiagen, Germany), the reaction conditions were as follows: 95°C for 3 min; 39 cycles at 95°C for 10 s, 60°C for 10 s, and 72°C for 30 s; and 95°C for 10 min, 65°C for 5 s, 95°C for 5 s. The sequences of the primers used for qPCR were shown in Table 2. Results were calculated and represented using the 2^®–ΔΔCT^ method.

**Table 2:** Primers used for real-time PCR.

Gene		Primer sequence (5′–3′)	Accession numbers
ZO-1	F	GTCCAGAATCTCGGAAAAGTGCC	AJ318101
	R	CTTTCAGCGCACCATACCAACC	
Occludin	F	CAGGTGCACCCTCCAGATTG	NM_001163647
	R	TATGTCGTTGCTGGGTGCAT	
β-actin	F	CAGGTCATCACCATCGGCAACG	NM_001199954.3
	R	GACAGCACCGTGTTGGCGTAGAGGT	

### Intestinal flora detection and analysis

We took the colon contents and extract the genomic DNA of the sample used the Cetyltrimethylammonium Bromide (CTAB) method, detected DNA purity and concentration by 1.5% agarose gel electrophoresis, the V3 and V4 regions of 16 s of 16 s DNA are known as templates. PCR amplification was carried out with specific primers with Barcode and high-fidelity enzymes, and the PCR products were electrophoretically detected with 2% agarose gel, then, the qualified fragments were recovered by the gel recovery kit provided by Qiagen Company, and sent to Beijing Novegene Technology Co., Ltd. (Beijing, China) for 16S rRNA gene sequencing.

### Statistical analysis

The original data were organized in Excel 2012. Data was analyzed using the one-way ANOVA and Tukey’s honestly significant difference test in GraphPad Prism 9.3, in addition, the diarrhea rates of piglets were compared with use of chi-square analysis, observations and expectations are expressed in percentage form (%) and the chi square value is 11.91, and microbial data was used nonparametric estimation to analysis. All the table data were expressed as means ± SD, and the figure data were expressed as means ± SEM. *p*-values <0.05 were considered significant.

## Results

### Effects of dietary CAP on growth performance and diarrhea rate in nursery piglets

The effect of dietary CAP on the growth performance of nursery Hainan piglets is presented in Table 3. Compared with the CON group, the diarrhea rate had significantly decreased (*p* < 0.05), and the ADG had increased by 8.01% and FRC decreased by 9.87% in the AC group, but there have no significant differences (*p* > 0.05). The FCR was significantly lower in 500 mg/kg CAP group than in that of the CON and the 1,000 mg/kg CAP groups (*p* < 0.05). Moreover, compared with that in the AC group, the FCR was decreased by 7.62% in the 500 mg/kg CAP group, although not significantly (*p* > 0.05). Additionally, the diarrhea rate of piglets in the 250 mg/kg and 500 mg/kg CAP groups was significantly decreased compared with the CON group (by 49.37 and 45.87%, respectively; *p* < 0.05); however, no significant differences were observed relative to the AC group (*p* > 0.05).

**Table 3:** Effects of dietary cecropin antimicrobial peptide (CAP) supplementation on growth performance and diarrhea rate in nursery piglets.

Item^1^	CON^2^	CAP treatment			AC^3^	*p*-value
		250 mg/kg	500 mg/kg	1,000 mg/kg		
Initial weight, kg	13.31 ± 0.21	13.45 ± 0.29	13.31 ± 0.24	13.29 ± 0.22	13.19 ± 0.08	0.601
Final weight, kg	22.95 ± 0.70^ab^	23.50 ± 1.03^ab^	24.23 ± 1.00^a^	21.25 ± 0.70^b^	23.38 ± 1.09^ab^	0.005
ADG, g	235.90 ± 10.51^b^	251.30 ± 20.05^ab^	291.40 ± 35.37^a^	231.79 ± 10.53^b^	254.80 ± 28.37^ab^	0.027
ADFI, g	549.30 ± 7.76	551.80 ± 7.18	533.10 ± 20.02	536.30 ± 9.16	530.70 ± 20.46	0.875
FCR	2.33 ± 0.08^a^	2.21 ± 0.19^ab^	1.94 ± 0.22^b^	2.36 ± 0.13^a^	2.10 ± 0.16^ab^	0.016
Diarrhea rate, %	8.85 ± 0.62^a^	4.48 ± 2.30^b^	4.79 ± 2.97^b^	7.49 ± 2.82^ab^	4.27 ± 2.29^b^	0.018

In the same row, values with different superscript lowercase letters differ significantly (*p* < 0.05). Data are presented as means ± SD (*n* = 4). ^1^ADG, average daily gain; ADFI, average daily feed intake; FCR, feed conversion ratio; ^2^CON, control; ^3^AC, antibiotic control.

### Effects of dietary CAP on serum biochemistry in nursery piglets

Here, compared with CON group, the serum level of ALB was increased and the serum level AST was significantly decreased in the AC group (*p* < 0.05, Table 4), and we found that piglets in the 500 mg/kg CAP group had higher serum levels of TP, ALB, and GLB (*p* < 0.05, Table 4) compared with the CON group. Compared with the AC group, the supplementation of 500 mg/kg CAP increased serum levels of TP and GLB in the piglets by 19.47% and 26.82%, respectively (*p* < 0.05, Table 4). Moreover, in the 500 mg/kg CAP group, serum AST was decreased by 36.17% compared with that in the CON group (*p* < 0.05, Table 4). However, no significant differences were detected in serum levels of ALT among the 5 groups of nursery Hainan piglets (*p* > 0.05, Table 4).

**Table 4:** Effects of dietary cecropin antimicrobial peptide (CAP) supplementation on serum biochemistry levels in nursery piglets.

Item^1^	CON^2^	CAP treatment			AC^3^	*p*-value
		250 mg/kg	500 mg/kg	1,000 mg/kg		
TP (g/L)	71.67 ± 1.47^b^	81.70 ± 10.76^ab^	92.50 ± 3.93^a^	77.13 ± 8.82^b^	77.42 ± 1.75^b^	0.006
GLB (g/L)	42.25 ± 2.60^b^	50.00 ± 8.32^ab^	52.38 ± 6.33^a^	47.00 ± 6.24^ab^	41.30 ± 3.25^b^	0.048
ALB (g/L)	28.60 ± 1.19^c^	32.75 ± 2.10^abc^	34.88 ± 1.79^ab^	29.30 ± 4.31^bc^	35.80 ± 4.52^a^	0.007
AST (U/L)	117.50 ± 10.41^a^	105.00 ± 17.80^ab^	75.00 ± 7.07^b^	99.00 ± 10.84^ab^	82.50 ± 22.17^b^	0.005
ALT (U/L)	46.00 ± 8.94	50.83 ± 8.61	41.25 ± 4.78	46.00 ± 2.24	41.00 ± 6.52	0.163

In the same row, values with different superscript lowercase letters differ significantly (*p* < 0.05). Data are presented as means ± SD (*n* = 6), the same as below. ^1^TP, total protein; GLB, globulin; ALB, albumin; AST, aspartate aminotransferase; ALT, alanine aminotransferase; ^2^CON, control; ^3^AC, antibiotic control.

### Effects of dietary CAP on serum immunoglobulin levels in nursery piglets

Serum immunoglobulin levels of weaned Hainan pigs showed that there had no significant difference in the serum level of IgG among the 5 groups (*p* > 0.05, Table 5). Compared with the CON group, serum IgM content had increased in the AC group, serum IgA content in the 500 mg/kg CAP group significantly increased by 141.90%, serum IgM content in the 250 mg/kg CAP and 500 mg/kg CAP groups had significantly increased by 17.71% and 21.15%, respectively (*p* < 0.05, Table 5), but the serum IgA content in the 250 mg/kg, 1,000 mg/kg CAP group and serum IgM in 1,000 mg/kg CAP group had no significantly increased (*p* > 0.05, Table 5). Compared with the AC group, serum IgA content in the 500 mg/kg CAP group was 16.51% higher, but there was no significant difference (*p* > 0.05, Table 5).

**Table 5:** Effects of dietary cecropin antimicrobial peptide (CAP) supplementation on serum immunoglobulin levels in nursery piglets.

Itema^1^	CON^2^	CAP treatment			AC^3^	*p*-value
		250 mg/kg	500 mg/kg	1,000 mg/kg		
IgA (μg/mL)	4.20 ± 0.63^b^	5.74 ± 1.21^b^	10.16 ± 2.84^a^	8.00 ± 1.26^ab^	8.72 ± 2.95^ab^	0.003
IgG (g/L)	8.29 ± 1.19	7.83 ± 0.65	7.92 ± 0.41	7.04 ± 1.52	7.50 ± 0.25	0.374
IgM (μg/mL)	18.58 ± 1.04^b^	21.87 ± 1.39^a^	22.51 ± 1.40^a^	20.75 ± 1.00^ab^	22.15 ± 1.59^a^	0.004

^1^Ig, immunoglobulin; ^2^CON, control; ^3^AC, antibiotic control.

### Effects of dietary CAP on the intestinal morphology of nursery piglets

**Figure 1:**
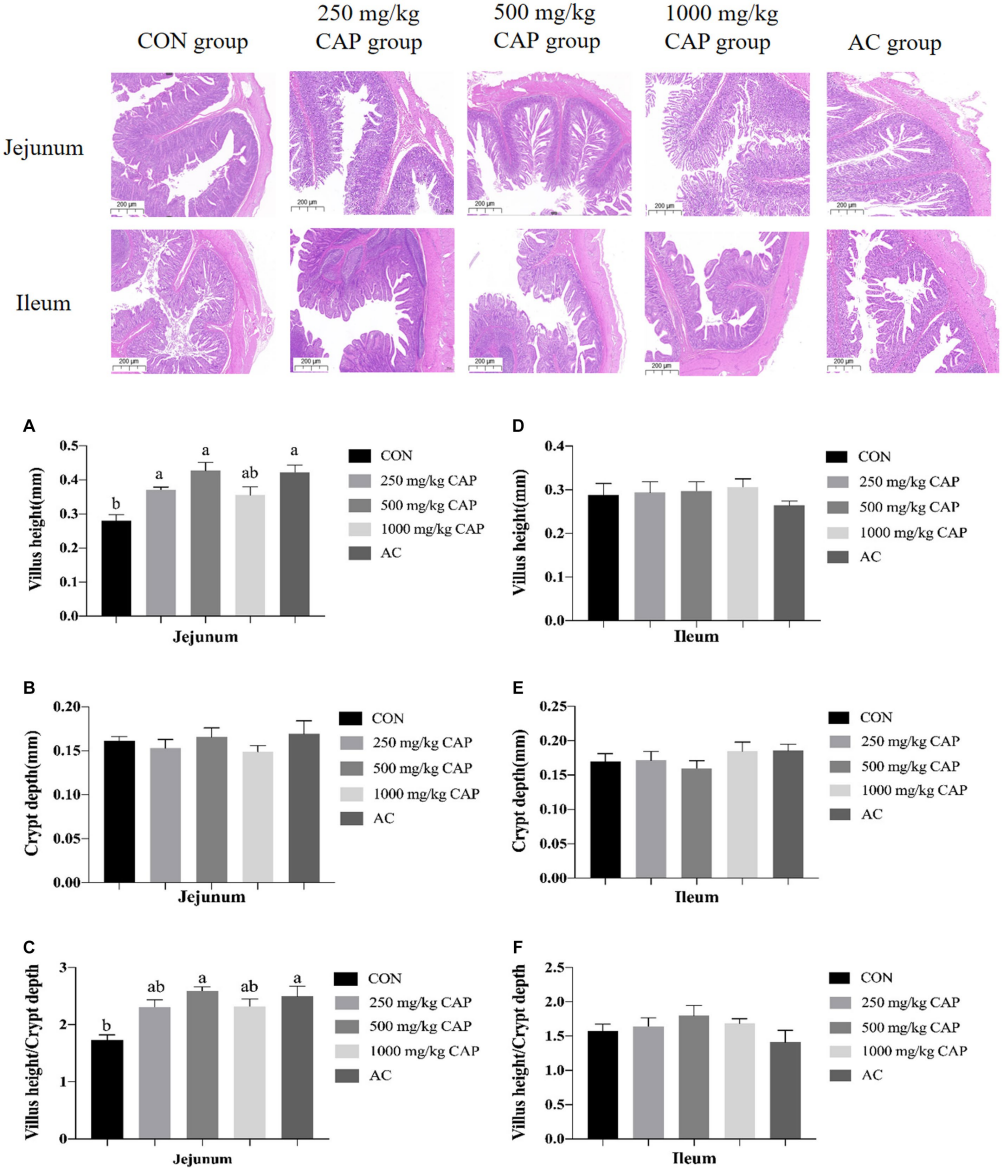
Effects of dietary cecropin antimicrobial peptide (CAP) supplementation on intestinal morphology in nursery piglets. **(A)** Jejunum villus height. **(B)** Jejunum crypt depth. **(C)** Jejunum villus height/crypt depth. **(D)** Ileum height. **(E)** Ileum crypt depth. **(F)** Ileum villus height/crypt depth. Data are presented as means ± SEM (*n* = 6); mean values with different letters indicate significant differences (*p* < 0.05).

The intestinal morphology of nursery Hainan piglets was shown in Figure 1. Compared with the CON group, the jejunal villus height was higher in the 250 mg/kg, 500 mg/kg CAP and AC groups (*p* < 0.05, Figure 1A); and there had no marked differences of jejunal crypt depth among the 5 groups (*p* > 0.05, Figure 1B), while the jejunal V/C ratio was significantly higher in the 500 mg/kg CAP and AC group than in the CON group (a 48.65% increase; *p* < 0.05, Figure 1C). No marked differences in villus height, crypt depth, or V/C ratio were found in the ileum among the 5 dietary treatment groups (*p* > 0.05, Figures 1D–F).

### Effects of dietary CAP on the pH of the gastrointestinal contents in nursery piglets

There had no significant differences in the pH value of the gastrointestinal contents between the control groups (CON and AC) and the 3 CAP supplementation groups (250, 500, and 1,000 mg/k, Table 6) (*p* > 0.05).

**Table 6:** Effects of dietary cecropin antimicrobial peptide (CAP) supplementation on the pH of the gastrointestinal contents in nursery piglets.

Item	CON	CAP treatment			AC	*p*-value
		250 mg/kg	500 mg/kg	1,000 mg/kg		
Stomach pH	2.92 ± 0.56	2.60 ± 0.46	2.61 ± 0.33	3.09 ± 0.59	3.21 ± 0.60	0.371
Duodenum pH	5.77 ± 0.40	5.72 ± 0.34	5.28 ± 0.73	5.54 ± 0.39	5.72 ± 0.44	0.109
Jejunum pH	5.76 ± 0.42	5.95 ± 0.37	5.70 ± 0.29	5.70 ± 0.31	5.78 ± 0.45	0.466
Ileum pH	6.17 ± 0.58	6.02 ± 0.59	5.94 ± 0.39	5.87 ± 0.42	6.05 ± 0.47	0.622

CON, control; AC, antibiotic control.

### Dietary CAP modulated intestinal tract integrity in nursery piglets

Compared with the CON group, serum *D*-lactic acid concentration and DAO activity were significantly reduced in the 500 mg/kg CAP group (by 31.16% and 54.83%, respectively) and AC group (by 13.04% and 43.31%, respectively; *p* < 0.05, Table 7); however, no differences were seen compared with the AC group (*p* > 0.05, Table 7). Furthermore, DAO activity was significantly lower in the 250 mg/kg and 1,000 mg/kg CAP groups than in the CON group, with reduced of 45.83% and 25.22%, respectively (*p* < 0.01, Table 7).

**Table 7:** Effects of dietary cecropin antimicrobial peptide (CAP) supplementation modulated intestinal tract integrity in nursery piglets.

Item^1^	CON^2^	CAP treatment			AC^3^	*p*-value
		250 mg/kg	500 mg/kg	1,000 mg/kg		
D-lactic acid (μg/mL)	0.69 ± 0.05^a^	0.63 ± 0.08^a^	0.47 ± 0.06^b^	0.45 ± 0.06^a^	0.60 ± 0.06^b^	<0.01
DAO (pg/mL)	40.43 ± 1.78^a^	21.90 ± 4.48^bc^	18.26 ± 3.71^c^	30.23 ± 5.55^b^	22.92 ± 4.21^bc^	<0.01

^1^DAO, diamine oxidase; ^2^CON, control; ^3^AC, antibiotic control.

### Effects of dietary CAP on the relative mRNA expressions of the tight junction gene in nursery piglets

**Figure 2:**
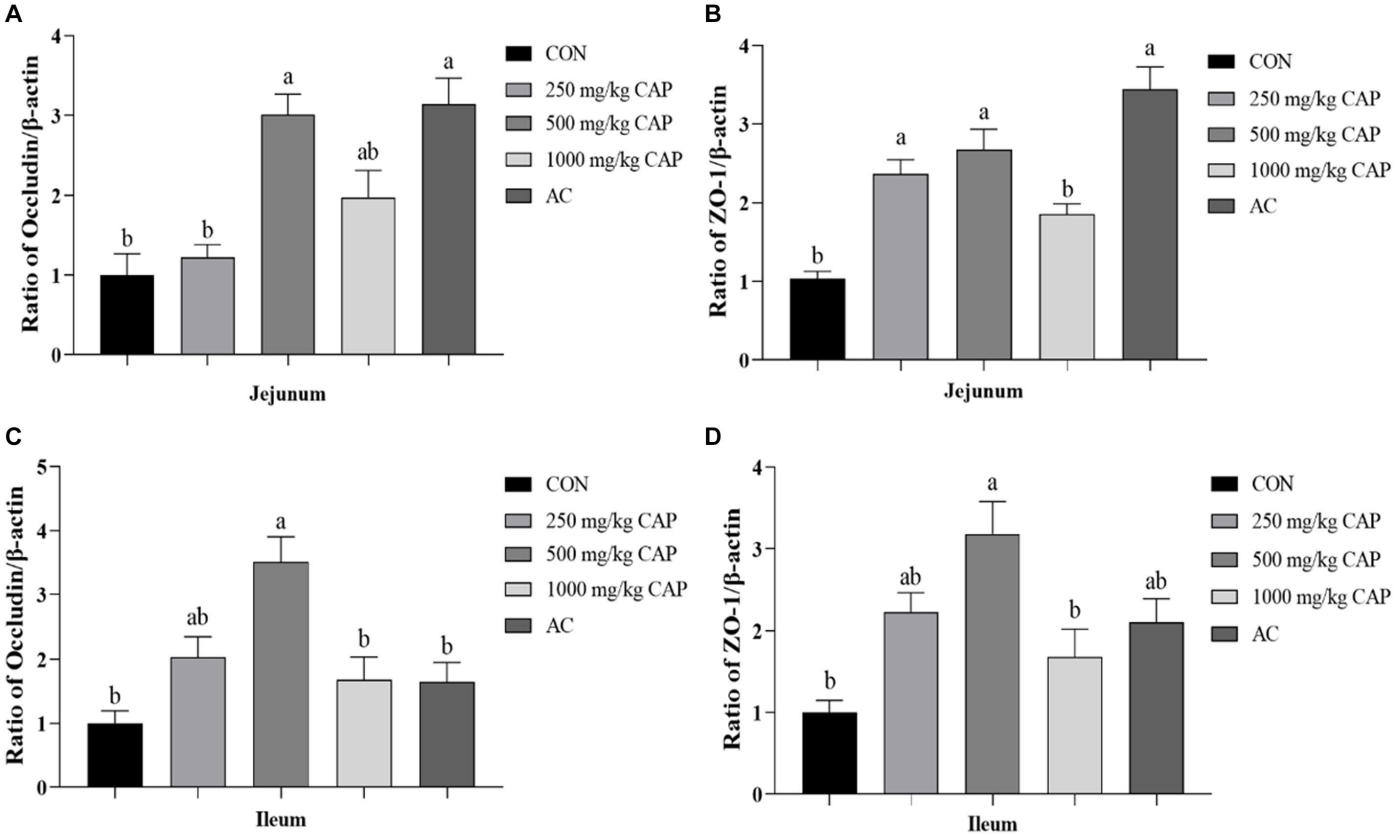
Effects of dietary cecropin antimicrobial peptide (CAP) supplementation on intestinal tight junction in nursery piglets. **(A*–*D)** The mRNA expression levels of genes associated with intestinal barrier function in nursery Hainan piglets in the different groups. **(A)** Relative expression of occludin mRNA in jejunum. **(B)** Relative expression of ZO-1 mRNA in jejunum. **(C)** Relative expression of occludin mRNA in ileum. **(D)** Relative expression of ZO-1 mRNA in ileum. Data are presented as means ± SEM (*n* = 6); mean values with different letters indicate significant differences (*p* < 0.05).

Compared with the CON group, the mRNA expression level of occludin had significantly increased in the 500 mg/kg CAP and AC groups of the jejunum (*p* < 0.05, Figure 2A), moreover, the occludin mRNA expression level in the 500 mg/kg CAP group was higher than that in the AC group, although the difference was not significant (*p* > 0.05, Figure 2A). Meanwhile, the occludin mRNA expression level in ileal tissue was significantly upregulated in the 500 mg/kg CAP group compared with that in the CON and AC groups (*p* < 0.05, Figure 2C). Additionally, the occludin mRNA expression level was higher of ileal tissue in the 250 mg/kg CAP group than in the CON group, but the difference was not significant (*p* > 0.05, Figure 2C). Compared with the CON group, the ZO-1 mRNA expression level was significantly increased in the jejunum of 250, 500 mg/kg CAP and AC groups and in ileum of the 500 mg/kg CAP group (*p* < 0.05, Figures 2B–D), but compared with AC group, the ZO-1 mRNA expression level of jejunum in the 1,000 mg/kg CAP group was significantly reduced (*p* < 0.05, Figure 2B).

### Effect of dietary CAP on the intestinal microflora of nursery piglets

**Figure 3:**
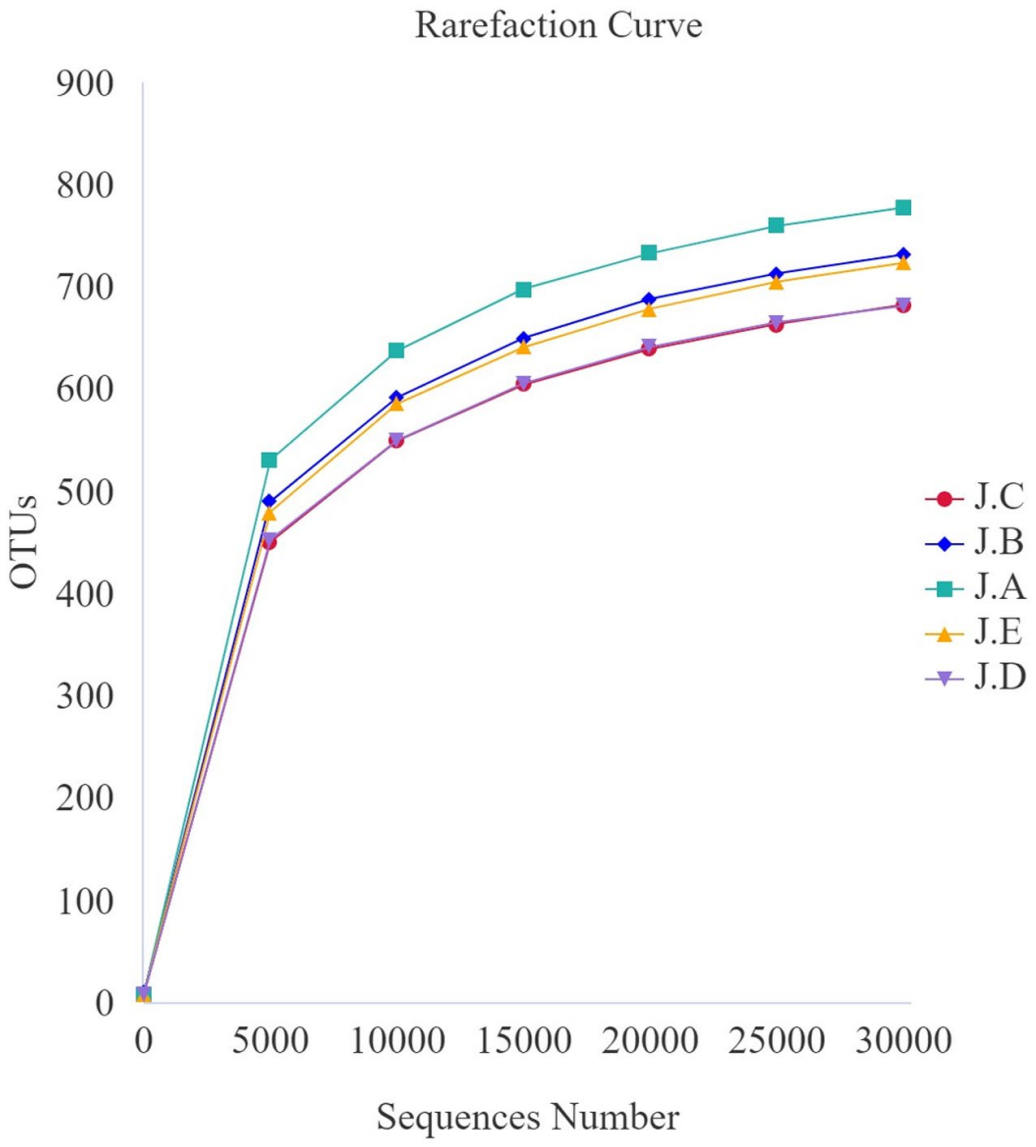
Colonic microbial dilution curve for nursery piglets. (J.A) negative control (NC) group, (J.B) 250 mg/kg cecropin antimicrobial peptide (CAP) supplementation group, (J.C) 500 mg/kg CAP supplementation group, and (J.D) 1,000 mg/kg CAP supplementation group, and (J.E) antibiotic control (AC) group. The same as below (*n* = 6).

The dilution curve for each sample and that of each group tended to be flat, indicating that the amount of sequencing data was reasonable (Figure 3). Additionally, as shown in the alpha-diversity index in Table 8, the Good’s coverage of each group was greater than 99.8%. Observed species and the Chao1 index of the 500 mg/kg CAP group were significantly lower than those of the CON group (*p* < 0.05, Table 8); however, no differences in Shannon and Simpson’s indices were found among the 5 groups (*p* > 0.05, Table 8).

**Table 8:** The alpha-diversity index of nursery Hainan piglets in the different treatment groups.

Item	CON	CAP treatment			AC	*p*-value
		250 mg/kg	500 mg/kg	1,000 mg/kg		
Goods coverage, %	99.84 ± 0.02	99.84 ± 0.05	99.85 ± 0.02	99.85 ± 0.01	99.83 ± 0.02	0.289
Chao1	808.60 ± 40.64^a^	742.50 ± 38.42^ab^	722.30 ± 40.47^b^	742.10 ± 48.07^ab^	760.20 ± 33.84^ab^	0.045
Observed species	777.50 ± 43.94^a^	728.70 ± 69.38^ab^	679.70 ± 49.55^b^	701.8 ± 48.57^ab^	701.60 ± 32.28^ab^	0.043
Shannon index	6.29 ± 0.32	5.93 ± 0.15	5.76 ± 0.61	5.92 ± 0.71	6.03 ± 0.46	0.191
Simpson’s index	0.94 ± 0.02	0.95 ± 0.02	0.93 ± 0.03	0.92 ± 0.05	0.94 ± 0.04	0.361

CON, control; AC, antibiotic control.

**Figure 4:**
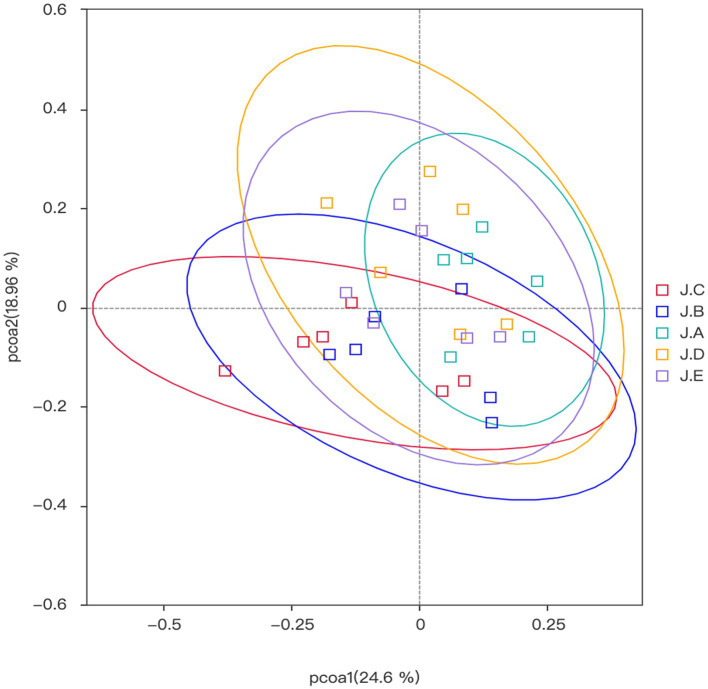
β diversity of the colonic microbiota (*n* = 6).

Beta diversity analysis is mainly used to compare the differences in the overall structure of microbial communities among different samples, each point in the graph represents a sample, and samples from the same group are represent the same color. If the sample distance is closer, it indicates that the species composition structure is more similar. Therefore, samples with high similarity in community structure tend to gather together, while samples with significant differences in community structure will be far apart. In this research, the colon microbial community structure of the NC group, 1,000 mg/kg CAP group, and PC group was relatively similar, while the colon microbial community structure of the 250 mg/kg and 500 mg/kg CAP groups was relatively similar (Figure 4).

As shown in Figure 3A, the most abundant bacteria in the colonic digesta of the piglets at the phylum level were Firmicutes, Bacteroidota, and Proteobacteria. Compared with the CON group, the relative abundance of *Lactobacillus* and *Limoslactobacillus* was significantly higher in the AC group (*p* < 0.05, Table 9). Additionally, the relative abundance of Firmicutes was significantly higher in the 500 mg/kg CAP group than in the CON group (*p* < 0.05, Table 9), while that of Proteobacteria was significantly lower in the 250 mg/kg CAP group than in the CON group (*p* < 0.05, Table 9). At the genus level, we found that *Lactobacillus*, *Streptococcus*, *Prevotella*_9, *Escherichia*–*Shigella*, and *Limosilactobacillus* were the dominant genera in all the groups (Figure 3B); moreover, the relative abundance of *Lactobacillus* in the 500 mg/kg CAP group was significantly higher than that in the CON group and AC group (*p* < 0.05, Table 9). The relative abundance of *Limoslactobacillus* was significantly higher in the 500 mg/kg CAP group than in the CON group (*p* < 0.05, Table 9), while that of *Streptococcus* and *Escherichia*–*Shigella* was significantly lower (*p* < 0.05, Table 9).

**Table 9:** Relative species abundance at the phylum and genus levels.

Items	CON	CAP treatment			AC	*p*-value
		250 mg/kg	500 mg/kg	1,000 mg/kg		
**Phylum level, %**						
Firmicutes	50.36 ± 2.72^b^	57.96 ± 6.36^ab^	69.44 ± 10.57^a^	60.14 ± 5.17^ab^	63.24 ± 4.57^ab^	0.015
Bacteroidota	29.45 ± 6.44	32.36 ± 8.90	22.45 ± 8.04	25.51 ± 8.08	26.94 ± 5.48	0.312
Proteobacteria	9.30 ± 1.94^a^	2.68 ± 1.99^b^	8.81 ± 1.75^a^	6.30 ± 3.61^ab^	5.72 ± 2.57^ab^	0.028
**Genus level, %**						
*Lactobacillus*	4.97 ± 2021^c^	16.55 ± 6.87^bc^	31.63 ± 9.06^a^	14.00 ± 4.65^bc^	17.24 ± 8.13^b^	0.002
*Limosilactobacillus*	0.92 ± 0.25^b^	3.11 ± 0.91^a^	3.37 ± 1.58^a^	4.22 ± 0.31^a^	3.44 ± 1.03^a^	0.014
*Prevotella*_9	4.36 ± 2.33	5.09 ± 1.63	3.46 ± 2.01	6.54 ± 3.76	4.42 ± 3.09	0.675
*Streptococcus*	19.07 ± 4.02^A^	10.24 ± 3.41^abc^	6.81 ± 1.45^c^	21.68 ± 7.15^a^	14.02 ± 3.94^a^	0.006
*Escherichia–Shigella*	2.35 ± 1.26^a^	0.88 ± 0.51^b^	0.75 ± 0.32^b^	1.41 ± 1.14^ab^	1.72 ± 1.08^ab^	0.047

CON, control; AC, antibiotic control.

## Discussion

After weaning, piglets can suffer from stress due to changes in feed intake, which may further cause diarrhea, growth retardation, and a decline in production performance. Over recent years, numerous studies have confirmed that dietary supplementation with AMPs can promote the development of the intestinal mucosa, improve intestinal morphology, and increase the weight of animals (Bao et al., 2009; Wu et al., 2012; Feng et al., 2020). Here, we found that nursery Hainan piglets fed diets containing 500 mg/kg CAP had greater ADG and FCR (*p* < 0.05, Table 3) compared with the CON group, but the addition levels of 250 mg/kg and 1,000 mg/kg had no significant effects on ADG and FCR (*p* > 0.05, Table 3). Yoon et al. (2013) observed an improvement in ADG and FCR in weanling piglets (Landrace × Yorkshire × Duroc) fed diets supplementation with 60 mg/kg hybrid AMP (cecropin A-magainin2). Another study showed that dietary supplementation with 1,000 mg/kg immobilized AMP was significantly increased the ADG and reduced FCR of weanling piglets (Landrace × Yorkshire × Duroc) (Liu et al., 2022). These studies indicate that AMPs in the diets of weaning piglets improved the growth performance, but due to different breeds of piglets and different types of AMP, the optimal dosage of antimicrobial peptides is also different.

Diarrhea is a common and frequently occurring disease among piglets. The digestive system of piglets is not mature both before and immediately after weaning, and the overall digestive function of the animals is relatively weak at this stage. After weaning, stress resulting from abrupt changes in dietary structure and feeding environment, among other causes, is very likely to cause intestinal barrier destruction, intestinal dysbiosis and immune decline in weaned piglets, leading to diarrhea (Ren et al., 2022). Post-weaning diarrhea is caused by bacteria such as *E. coli* (Huang et al., 2018), *Salmonella* (Kylla et al., 2019), and *Clostridium perfringens* (Songer, 2010) and represents one of the most serious problems facing the swine industry worldwide. In this study, we found that compared with the CON group, the diarrhea rate was significantly reduced in nursery Hainan piglets fed diets containing 250 mg/kg and 500 mg/kg CAP (*p* < 0.05, Table 3), and the effect was equivalent to that seen in the antibiotic supplementation group. Wu et al. (2021) showed that the AMP GW-Q4 had strong antibacterial activity against enterotoxigenic *E. coli*, and the authors reported that GW-Q4 might be a promising candidate for the treatment of diarrhea in weaned piglets caused by this bacterium. Meanwhile, Liang et al. (2022) showed that the AMP gloverin2 exhibited desirable antimicrobial activity against 3 indicator bacteria associated with piglet diarrhea (*E. coli*, *Salmonella* Derby, and *C. perfringens*) and also displayed marked potential for the treatment of this condition in piglets. *Escherichia* could cause diarrhea in humans, cows, and pigs (Bin et al., 2018), and in our study showed that CAP significantly reduced the abundance of *Escherichia–Shigella*. Therefore, the above results indicated that AMP could reduce piglet diarrhea by inhibiting the reproduction of harmful bacteria. But there were no differences in diarrhea rates between nursery Hainan piglets of the CON group and 1,000 mg/kg CAP group (*p* > 0.05, Table 3), the reason may be that 1,000 mg/kg CAP fed for more than 4 weeks inhibited lymphocyte proliferation, promoted apoptosis, reduced piglets’ immunity, and thus weakened their ability to resist pathogens (Ren et al., 2015). Moreover, *Streptococcus* contains multiple pathogenic bacteria, infected the intestine will cause damage to the intestinal mucosa and cause diarrhea in piglets (Ferrando and Schultsz, 2016), the higher relative abundance of *Streptococcus* may be one of the reasons for the higher diarrhea rate of piglets in the 1,000 mg/kg CAP group. However, further investigation is needed to investigate the reasons for this.

Serum biochemical indicators reflect the metabolic and health status of the body to varying degrees. Serum TP include ALB and GLB, which are synthesized by the liver. Serum TP and ALB concentrations can reflect the status of protein metabolism, at least to some extent, while the serum GLB level is closely related to the immune status (Geng et al., 2021). Immunoglobulin G participates in the humoral immune response, while IgM and IgA are involved in the mucosal immune response (Megha and Mohanan, 2021). Our results showed that, although serum AST was decreased of 500 mg/kg CAP group compared with that in the CON group, all groups of piglet serum AST were within the normal physiological range (Feldman et al., 2000); compared with the CON group, the 500 mg/kg CAP group had higher serum IgA and IgM levels (*p* < 0.05, Table 5). Moreover, serum IgA concentrations in the 500 mg/kg CAP group were greater than those in the AC group (*p* > 0.05, Table 5). Additionally, the increased serum levels of IgA, and IgM implied that the dietary inclusion of 500 mg/kg CAP enhanced the immunity of the nursery Hainan piglets. Wu et al. (2012) demonstrated that the addition of cecropins AD to the diets of weaned piglets (Landrace × Yorkshire) improved the levels of serum IgA and IgG. It has also been shown that the antimicrobial peptide LL-37 can promote antigen-specific immune responses in mice by upregulating the levels of soluble IgA and serum IgG, thereby enhancing mucosal and systemic immunity (Kim et al., 2015). Our findings were consistent with these reports, which indicated that CAP could promote mucosal immunity in nursery Hainan piglets, possibly because that dietary AMP increased both the number and the proliferative ability of T cells and significantly improved immune function in weaned piglets (Ren et al., 2015), additionally, at the dosage of 500 mg/kg, the effect was superior to that of the AC group.

**Figure 5:**
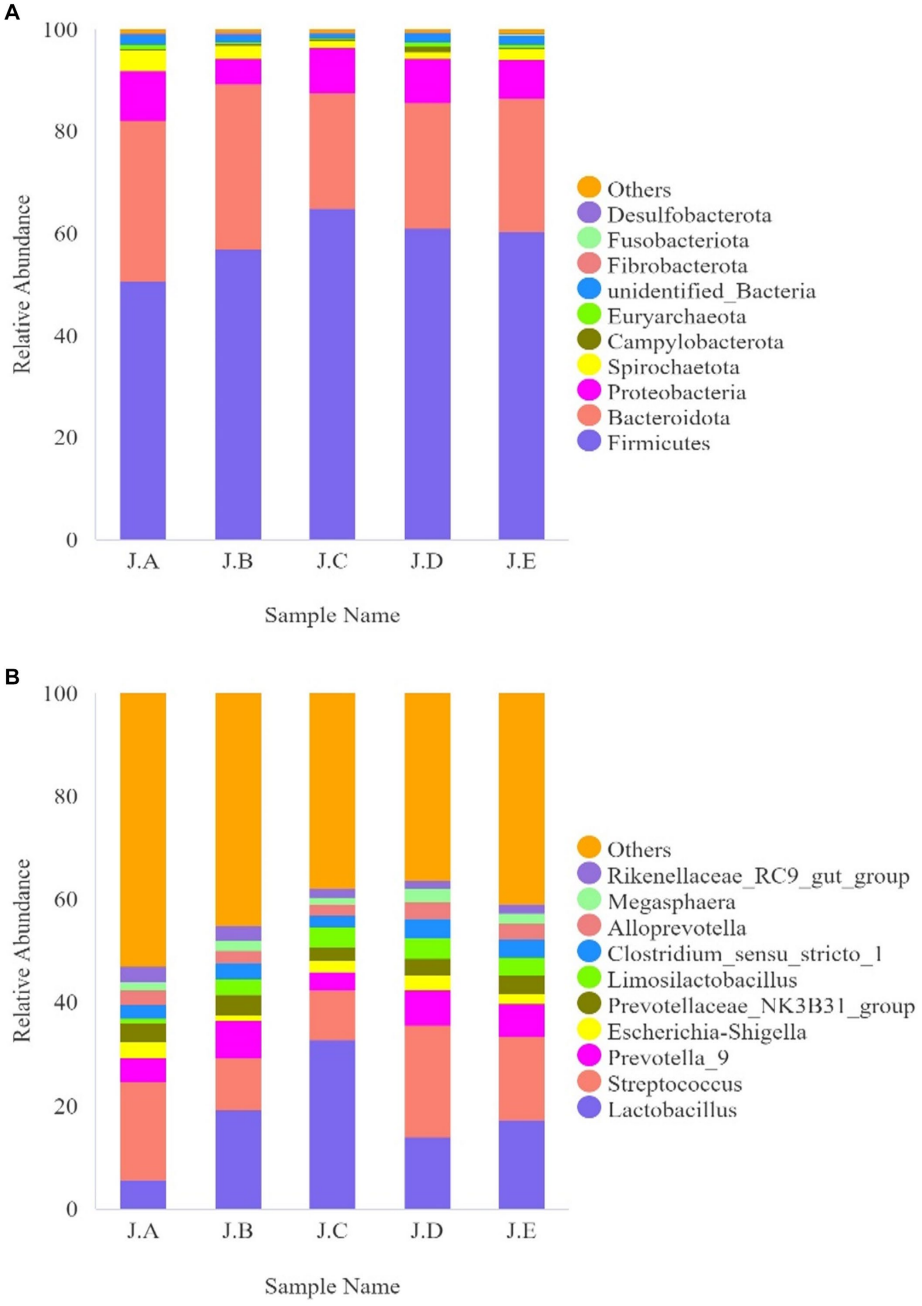
Effects of dietary cecropin antimicrobial peptide (CAP) supplementation on intestinal microflora abundance in nursery piglets. **(A)** Histogram of the top 10 species with the greatest relative abundance at the phylum level. **(B)** Histogram of the top 10 species with the greatest relative abundance at the genus level (*n* = 6).

Growth performance of piglet is highly associated with intestinal functions. The small intestine is a crucial digestive and absorptive organ for nutrients. The V/C ratio represents the absorptive capacity of the small intestine, and the higher ratio, the better the intestinal absorption capacity (Zhang et al., 2021). Tight junctions play a key role in the maintenance of the structural and functional integrity of the intestinal mucosa. They are composed of transmembrane cell adhesion molecules, including claudin, occludin, and the peripheral membrane protein ZO-1 (Suzuki, 2020). The primary function of tight junctions is to prevent the invasion of inflammatory mediators into the systemic circulation by allowing only ions and soluble small-molecule substances to traverse the intestinal barrier (Suzuki, 2013). Serum *D*-lactic acid and DAO levels are sensitive markers for detecting intestinal permeability and tissue injury. When the intestine is damaged, intestinal permeability increases, leading to an increase in serum *D*-lactic acid and DAO levels (Wang et al., 2022). In our study, compared with the CON group, nursery Hainan piglets fed a diet containing 500 mg/kg CAP exhibited a higher V/C ratio and greater villus length in the jejunum (*p* < 0.05, Figure 2), lower serum *D*-lactic acid and DAO levels (*p* < 0.05, Table 6), and increased expression of ZO-1 and occludin mRNA (*p* < 0.05, Figure 5). Wen and He (2012) demonstrated that the addition of cecropins to the diets of broilers promoted the growth of intestinal villi and improved intestinal morphology. It was also reported that supplementation of magainin II-cecropin B, a hybrid AMP, increased the length of ileal villi, the V/C ratio, and the expression level of intestinal ZO-1, claudin-1, and occludin mRNA in mice (Zhang et al., 2018). In a porcine jejunum epithelial cell (IPEC-J2) intestinal barrier model, cecropin A was observed to modulate ZO-1 and occludin protein expression and enhance barrier function by suppressing the MEK/ERK pathway (Zhai et al., 2018). It indicated that the improvement of intestinal morphology and intestinal tight junctions by CAP supplementation may be related to the suppression of MEK/ERK pathway by cecropins. However, this possibility requires further experimental verification. Study showed that the improvement of intestinal morphology and intestinal barrier could promote the digestion and absorption of nutrients, thus improving production performance (Oladokun et al., 2021). It also can explain why CAP could improve growth performance in nursery Hainan piglets.

Gut microbes also play an active role in maintaining intestinal health in weaned piglets (Xiang et al., 2021). The gut microbiota barrier is an important barrier against the invasion of exogenous pathogens and harmful substances (Amoroso et al., 2020). After weaning, piglets are prone to intestinal stress, which can lead to disturbances in the intestinal microbiota and reduced growth (Wang et al., 2017). In this study, compared with CON group, at the phylum levels, the relative abundance of Firmicutes was increased in the colons of 500 mg/kg CAP group, at the genus levels, *Lactobacillus* and *Limoslactobacillus* was increased in the colons of 500 mg/kg CAP group, whereas that of *Streptococcus* and *Escherichia*–*Shigella* was reduced (*p* < 0.05, Table 9); meanwhile, observed species and the Chao1 index of colonic microorganisms were also decreased (*p* < 0.05), possibly because that dietary supplementation of 500 mg/kg CAP significantly inhibited the growth and reproduction of harmful bacteria. Studies have shown that *Lactobacillus* can enhance growth performance and intestinal immunity in piglets (Xin et al., 2020; Yang et al., 2020) while *Limoslactobacillus* was reported to enhance gut integrity and immunomodulation, and attenuate hepatic disorders (Abuqwider et al., 2022). And *Escherichia*–*Shigella* belongs to Proteobacteria, including Gram-negative bacteria that produce LPS, and its increase will cause intestinal inflammation, it reported that the relative abundance of *Escherichia-Shigella* in the cecum of colitis model mice was more than 10 times higher than that of healthy mice (Peng et al., 2020). *Streptococcus* contains multiple pathogenic bacteria, colonization in the intestine can cause damage to the intestinal mucosa and cause diarrhea in piglets (Ferrando and Schultsz, 2016). Therefore, the abundance of *Lactobacillus* increased in the colon of piglets while decreasing that of *Escherichia–Shigella* and *Streptococcus* are also the reason that CAP supplementation improved intestinal morphology and intestinal barrier function to promote growth performance. In this study, piglets in the 500 mg/kg CAP group had greater ADG and relative abundance of *Lactobacillus* relative to the other 4 groups. This was in agreement with a study showing that the proportion of the *Lactobacillus* genus was positively related to BW and ADG in broilers (Chen and Yu, 2020). Meanwhile, there have a study shown that reduction of stomach pH can have beneficial effects on both growth performance and microbiota of weaning piglets (Lingbeek et al., 2021), in this study, no marked differences in the pH value of the gastrointestinal contents were observed between the control groups (CON and AC) and the 3 CAP supplementation groups (*p* > 0.05, Table 6), it indicated that dietary supplementation of CAP has no improved intestinal flora structure by regulating intestinal PH. These observations indicated that the dietary supplementation of 500 mg/kg CAP promoted growth performance and intestinal tight junction in nursery Hainan piglets through gut microbiota optimization, and its effect was better than that of the antibiotic group. The comprehensive results showed that cecropins could reduce diarrhea rate and promote growth performance of piglets by improving intestinal morphology, strengthening intestinal tight junction, and improving intestinal microbial structure.

Colistin sulfuric as antibiotic additives was often used as a feed additive in piglet breeding to improve intestinal health and reduce the diarrhea rate of piglets (Aden et al., 1969). And in this study, our results showed that the diarrhea rate had significantly decreased (*p* < 0.05), and the ADG had increased by 8.01% and FRC decreased by 9.87% in the AC group than that in the CON group, and compared with the CON group, the villus height, V/D, the mRNA expression level of occludin and ZO-1 were higher in the AC group (*p* < 0.05), those indicated that colistin sulfuric improved the intestinal health of piglets, reduced diarrhea rate, and had a certain growth promoting effect, it related to research results such as Peng et al. (2021). And in this study, CAP had the same effect, indicated that CAP has the potential to replace antibiotics in the treatment of piglet diarrhea.

## Conclusion

In summary, our data demonstrated that the dietary addition of CAPs can improve growth performance, reduce diarrhea and enhance immunity by improving intestinal flora structure, intestinal morphology, and intestinal tight junction. Cecropins have potential as an alternative to antibiotics in the diets of Hainan piglets, and providing a reference for the Chinese local piglet breeding industry.

## Data availability statement

The original contributions presented in the study are publicly available. This data can be found here: NCBI BioProject, PRJNA1080503.

## Ethics statement

The animal study was approved by Experimental Animal Ethics Committee of Animal Husbandry and Veterinary Research Institute, Hainan Academy of Agricultural Sciences (HNSYY20230203). The study was conducted in accordance with the local legislation and institutional requirements.

## Author contributions

KO: Conceptualization, Data curation, Formal analysis, Investigation, Software, Writing – original draft, Writing – review & editing. TC: Investigation, Writing – review & editing. RS: Data curation, Investigation, Project administration, Supervision, Writing – review & editing. YX: Conceptualization, Formal analysis, Writing – review & editing. QQ: Conceptualization, Investigation, Writing – review & editing. XL: Conceptualization, Writing – review & editing. JL: Data curation, Writing – review & editing. QL: Investigation, Methodology, Writing – review & editing. LW: Resources, Writing – review & editing.

## References

[ref1] Abuqwider J. Altamimi M. Mauriello G. (2022). *Limosilactobacillus reuteri* in health and disease. Microorganisms 10:522. doi: 10.3390/microorganisms10030522, PMID: 35336098 PMC8953724

[ref2] Aden D. P. Reed N. D. Underdahl N. R. Mebus C. A. (1969). Transferable drug resistance among enterobacteriaceae isolated from cases of neonatal diarrhea in calves and piglets. Appl. Microbiol. 18, 961–964. doi: 10.1128/am.18.6.961-964.1969, PMID: 4905699 PMC378176

[ref3] Amoroso C. Perillo F. Strati F. Fantini M. C. Caprioli F. Facciotti F. (2020). The role of gut microbiota biomodulators on mucosal immunity and intestinal inflammation. Cell 9:1234. doi: 10.3390/cells9051234 PMC7291275 32429359

[ref4] Bao H. She R. Liu T. Zhang Y. Peng K. S. Luo D.. (2009). Effects of pig antibacterial peptides on growth performance and intestine mucosal immune of broiler chickens. Poult. Sci. 88, 291–297. doi: 10.3382/ps.2008-00330, PMID: 19151342

[ref5] Benke S. N. Thulasiram H. V. Gopi H. N. (2017). Potent antimicrobial activity of lipidated short α,γ‐hybrid peptides. ChemMedChem 12, 1610–1615. doi: 10.1002/cmdc.201700370 28834198

[ref6] Bin P. Tang Z. Liu S. Chen S. Xia Y. Liu J.. (2018). Intestinal microbiota mediates enterotoxigenic *Escherichia coli*-induced diarrhea in piglets. BMC Vet. Res. 14:385. doi: 10.1186/s12917-018-1704-9, PMID: 30518356 PMC6282381

[ref7] Chen Y. C. Yu Y. H. (2020). *Bacillus licheniformis*-fermented products improve growth performance and the fecal microbiota community in broilers. Poult. Sci. 99, 1432–1443. doi: 10.1016/j.psj.2019.10.061, PMID: 32115030 PMC7587626

[ref9] Drider D. Boukherroub R. Le Devendec L. Belguesmia Y. Hazime N. Mourand G.. (2022). Impact of colistin and colistin-loaded on alginate nanoparticles on pigs infected with a colistin-resistant enterotoxigenic *Escherichia coli* strain. Vet. Microbiol. 266:109359. doi: 10.1016/j.vetmic.2022.109359, PMID: 35121303

[ref10] Feldman B. F. Zinkl J. G. Jain N. C. (2000). Schalm’s veterinary hematology. New Jersey: Wiley-Blackwell.

[ref11] Feng J. Wang L. Xie Y. Chen Y. Yi H. He D. (2020). Effects of antimicrobial peptide cathelicidin-BF on diarrhea controlling, immune responses, intestinal inflammation and intestinal barrier function in piglets with postweaning diarrhea. Int. Immunopharmacol. 85:106658. doi: 10.1016/j.intimp.2020.106658, PMID: 32531710

[ref12] Ferrando M. L. Schultsz C. (2016). A hypothetical model of host-pathogen interaction of *Streptococcus suis* in the gastro-intestinal tract. Gut Microbes 7, 154–162. doi: 10.1080/19490976.2016.1144008, PMID: 26900998 PMC4856463

[ref13] Geng A. L. Zhang Y. Zhang J. Zeng L. C. Chang C. Wang H. H.. (2021). Effects of light regime on the hatching performance, body development and serum biochemical indexes in Beijing You Chicken. Poult. Sci. 100:101270. doi: 10.1016/j.psj.2021.101270, PMID: 34237543 PMC8267589

[ref14] Gresse R. Chaucheyras-Durand F. Fleury M. A. van de Wiele T. Forano E. Blanquet-Diot S. (2017). Gut microbiota dysbiosis in postweaning piglets: understanding the keys to health. Trends Microbiol. 25, 851–873. doi: 10.1016/j.tim.2017.05.004, PMID: 28602521

[ref15] Guo C. Huang Y. Cong P. Liu X. Chen Y. He Z. (2014). Cecropin P1 inhibits porcine reproductive and respiratory syndrome virus by blocking attachment. BMC Microbiol. 14:273. doi: 10.1186/s12866-014-0273-8, PMID: 25403758 PMC4243277

[ref16] Hart G. K. Dobb G. J. (1988). Effect of a fecal bulking agent on diarrhea during enteral feeding in the critically ill. JPEN J. Parenter. Enteral. Nutr. 12, 465–468. doi: 10.1177/0148607188012005465, PMID: 3141642

[ref17] Huang Z. Kingsolver M. B. Avadhanula V. Hardy R. W. (2013). An antiviral role for antimicrobial peptides during the arthropod response to alphavirus replication. J. Virol. 87, 4272–4280. doi: 10.1128/JVI.03360-12, PMID: 23365449 PMC3624382

[ref18] Huang G. Li X. Lu D. Liu S. Suo X. Li Q.. (2018). Lysozyme improves gut performance and protects against enterotoxigenic *Escherichia coli* infection in neonatal piglets. Vet. Res. 49:20. doi: 10.1186/s13567-018-0511-4, PMID: 29463305 PMC5819691

[ref19] Jaber S. Iliev I. Angelova T. Nemska V. Sulikovska I. Naydenova E.. (2021). Synthesis, antitumor and antibacterial studies of new shortened analogues of (KLAKLAK)_2_-NH_2_ and their conjugates containing unnatural amino acids. Molecules 26:898. doi: 10.3390/molecules26040898, PMID: 33567789 PMC7915940

[ref20] Jensen M. S. Jensen S. K. Jakobsen K. (1997). Development of digestive enzymes in pigs with emphasis on lipolytic activity in the stomach and pancreas. J. Anim. Sci. 75, 437–445. doi: 10.2527/1997.752437x 9051467

[ref21] Kim S. H. Yang I. Y. Kim J. Lee K. Y. Jang Y. S. (2015). Antimicrobial peptide Ll-37 promotes antigen-specific immune responses in mice by enhancing Th17-skewed mucosal and systemic immunities. Eur. J. Immunol. 45, 1402–1413. doi: 10.1002/eji.201444988, PMID: 25655317

[ref22] Kwa A. Kasiakou S. K. Tam V. H. Falagas M. E. (2007). Polymyxin B: similarities to and differences from colistin (polymyxin E). Expert Rev. Anti Infect. Ther. 5, 811–821. doi: 10.1586/14787210.5.5.811, PMID: 17914915

[ref23] Kylla H. Dutta T. K. Roychoudhury P. Subudhi P. K. (2019). Coinfection of diarrheagenic bacterial and viral pathogens in piglets of northeast region of India. Vet. World 12, 224–230. doi: 10.14202/vetworld.2019.224-230, PMID: 31040562 PMC6460878

[ref24] Liang Q. Cao L. Zhu C. Kong Q. Sun H. Zhang F.. (2022). Characterization of recombinant antimicrobial peptide BMGlv2 heterologously expressed in *Trichoderma reesei*. Int. J. Mol. Sci. 23:10291. doi: 10.3390/ijms231810291, PMID: 36142214 PMC9499586

[ref25] Lingbeek M. M. Borewicz K. Febery E. Han Y. Doelman J. van Kuijk S. J. A. (2021). Short-chain fatty acid administration via water acidifier improves feed efficiency and modulates fecal microbiota in weaned piglets. J. Anim. Sci. 99:Skab307. doi: 10.1093/jas/skab307, PMID: 34679178 PMC8599185

[ref26] Liu X. Guo C. Huang Y. Zhang X. Chen Y. (2015). Inhibition of porcine reproductive and respiratory syndrome virus by cecropin D *in vitro*. Infect. Genet. Evol. 34, 7–16. doi: 10.1016/j.meegid.2015.06.021, PMID: 26102162

[ref27] Liu N. Ma X. Jiang X. (2022). Effects of immobilized antimicrobial peptides on growth performance, serum biochemical index, inflammatory factors, intestinal morphology, and microbial community in weaning pigs. Front. Immunol. 13:872990. doi: 10.3389/fimmu.2022.872990, PMID: 35422808 PMC9001916

[ref28] Luo C. C. Yin D. Y. Gao X. J. Li Q. Z. Zhang L. (2013). Goat mammary gland expression of cecropin B to inhibit bacterial pathogens causing mastitis. Anim. Biotechnol. 24, 66–78. doi: 10.1080/10495398.2012.745417, PMID: 23394371

[ref29] Megha K. B. Mohanan P. V. (2021). Role of immunoglobulin and antibodies in disease management. Int. J. Biol. Macromol. 169, 28–38. doi: 10.1016/j.ijbiomac.2020.12.073 33340621

[ref30] Montagne L. Cavaney F. S. Hampson D. J. Lallès J. P. Pluske J. R. (2004). Effect of diet composition on postweaning colibacillosis in piglets. J. Anim. Sci. 82, 2364–2374. doi: 10.2527/2004.8282364x, PMID: 15318736

[ref31] Nesa J. Sadat A. Buccini D. F. Kati A. Mandal A. K. Franco O. L. (2019). Antimicrobial peptides from *Bombyx mori*: a splendid immune defense response in silkworms. RSC Adv. 10, 512–523. doi: 10.1039/C9RA06864C 35492565 PMC9047522

[ref8] NY/T 65-2004. (2004). Pig Feeding Standards. Beijing: Standards Press of China.

[ref32] Oladokun S. Koehler A. Macisaac J. Ibeagha-Awemu E. M. Adewole D. I. (2021). *Bacillus subtilis* delivery route: effect on growth performance, intestinal morphology, cecal short-chain fatty acid concentration, and cecal microbiota in broiler chickens. Poult. Sci. 100:100809. doi: 10.1016/j.psj.2020.10.063, PMID: 33518343 PMC7936168

[ref33] Pane K. Sgambati V. Zanfardino A. Smaldone G. Cafaro V. Angrisano T.. (2016). A new cryptic cationic antimicrobial peptide from human apolipoprotein E with antibacterial activity and immunomodulatory effects on human cells. FEBS J. 283, 2115–2131. doi: 10.1111/febs.13725, PMID: 27028511

[ref34] Peng L. Gao X. Nie L. Xie J. Dai T. Shi C.. (2020). Astragalin attenuates dextran sulfate sodium (DSS)-induced acute experimental colitis by alleviating gut microbiota dysbiosis and inhibiting Nf-Κb activation in mice. Front. Immunol. 11:2058. doi: 10.3389/fimmu.2020.02058, PMID: 33042117 PMC7523281

[ref35] Peng C. Zuo S. Qiu Y. Fu S. Peng L. (2021). Determination of colistin in contents derived from gastrointestinal tract of feeding treated piglet and broiler. Antibiotics 10:422. doi: 10.3390/antibiotics10040422, PMID: 33921200 PMC8070394

[ref36] Ren W. Yu B. Yu J. Zheng P. Huang Z. Luo J.. (2022). Lower abundance of bacteroides and metabolic dysfunction are highly associated with the post-weaning diarrhea in piglets. Sci. China Life. Sci. 65, 2062–2075. doi: 10.1007/s11427-021-2068-6, PMID: 35467318

[ref37] Ren Z. H. Yuan W. Deng H. D. Deng J. L. Dan Q. X. Jin H. T.. (2015). Effects of antibacterial peptide on cellular immunity in weaned piglets. J. Anim. Sci. 93, 127–134. doi: 10.2527/jas.2014-7933, PMID: 25403191

[ref38] Salim H. M. Huque K. S. Kamaruddin K. M. Haque Beg A. (2018). Global restriction of using antibiotic growth promoters and alternative strategies in poultry production. Sci. Prog. 101, 52–75. doi: 10.3184/003685018X15173975498947 29467062 PMC10365203

[ref39] Sallum U. W. Chen T. T. (2008). Inducible resistance of fish bacterial pathogens to the antimicrobial peptide cecropin B. Antimicrob. Agents Chemother. 52, 3006–3012. doi: 10.1128/AAC.00023-08, PMID: 18474580 PMC2533505

[ref40] Songer J. G. (2010). Clostridia as agents of zoonotic disease. Vet. Microbiol. 140, 399–404. doi: 10.1016/j.vetmic.2009.07.003 19682805

[ref41] Su W. Gong T. Jiang Z. Lu Z. Wang Y. (2022). The role of probiotics in alleviating postweaning diarrhea in piglets from the perspective of intestinal barriers. Front. Cell Infect. Microbiol. 12:883107. doi: 10.3389/fcimb.2022.883107, PMID: 35711653 PMC9197122

[ref42] Sun T. Miao H. Zhang C. Wang Y. Liu S. Jiao P.. (2022). Effect of dietary *Bacillus coagulans* on the performance and intestinal microbiota of weaned piglets. Animal 16:100561. doi: 10.1016/j.animal.2022.100561, PMID: 35716416

[ref43] Suzuki T. (2013). Regulation of intestinal epithelial permeability by tight junctions. Cell Mol. Life Sci. 70, 631–659. doi: 10.1007/s00018-012-1070-x 22782113 PMC11113843

[ref44] Suzuki T. (2020). Regulation of the intestinal barrier by nutrients: the role of tight junctions. Anim. Sci. J. 91:E13357. doi: 10.1111/asj.13357, PMID: 32219956 PMC7187240

[ref45] Vanzolini T. Bruschi M. Rinaldi A. C. Magnani M. Fraternale A. (2022). Multitalented synthetic antimicrobial peptides and their antibacterial, antifungal and antiviral mechanisms. Int. J. Mol. Sci. 23:545. doi: 10.3390/ijms23010545, PMID: 35008974 PMC8745555

[ref46] Wang J. Han Y. Meng F. Zhao J. Zhou Z. Fan H. (2017). Fecal microbiota succession of piglets from birth to post-weaning by 454 pyrosequencing analysis. Trans. Tianjin Univ. 23, 211–220. doi: 10.1007/s12209-017-0045-2

[ref47] Wang G. Y. Shang D. Zhang G. X. Song H. Y. Jiang N. Liu H. H.. (2022). Qingyi decoction attenuates intestinal epithelial cell injury via the calcineurin/nuclear factor of activated T-cells pathway. World J. Gastroenterol. 28, 3825–3837. doi: 10.3748/wjg.v28.i29.3825, PMID: 36157544 PMC9367229

[ref48] Wen L. F. He J. G. (2012). Dose-response effects of an antimicrobial peptide, a cecropin hybrid, on growth performance, nutrient utilisation, bacterial counts in the digesta and intestinal morphology in broilers. Br. J. Nutr. 108, 1756–1763. doi: 10.1017/S0007114511007240, PMID: 22251659

[ref49] Wu K. C. Hua K. F. Yu Y. H. Cheng Y. H. Cheng T. T. Huang Y. K.. (2021). Antibacterial and antibiofilm activities of novel antimicrobial peptides against multidrug-resistant enterotoxigenic *Escherichia coli*. Int. J. Mol. Sci. 22:3926. doi: 10.3390/ijms22083926, PMID: 33920239 PMC8070514

[ref50] Wu S. Zhang F. Huang Z. Liu H. Xie C. Zhang J.. (2012). Effects of the antimicrobial peptide cecropin ad on performance and intestinal health in weaned piglets challenged with *Escherichia coli*. Peptides 35, 225–230. doi: 10.1016/j.peptides.2012.03.030, PMID: 22490448

[ref51] Xiang X. D. Deng Z. C. Wang Y. W. Sun H. Wang L. Han Y. M.. (2021). Organic acids improve growth performance with potential regulation of redox homeostasis, immunity, and microflora in intestines of weaned piglets. Antioxidants 10:1665. doi: 10.3390/antiox10111665, PMID: 34829536 PMC8615128

[ref52] Xiao J. (2011). Records of animal genetic resources in Hainan province. Haikou: Hainan press.

[ref53] Xin J. Zeng D. Wang H. Sun N. Zhao Y. Dan Y.. (2020). Probiotic *Lactobacillus johnsonii* Bs15 promotes growth performance, intestinal immunity, and gut microbiota in piglets. Probiotics Antimicrob. Proteins 12, 184–193. doi: 10.1007/s12602-018-9511-y, PMID: 30617949

[ref54] Xu X. Pan Y. Xu B. Yan Y. Yin B. Wang Y.. (2020). Effects of cortex phellodendri extract on post-weaning piglets diarrhoea. Vet. Med. Sci. 6, 901–909. doi: 10.1002/vms3.304, PMID: 32585771 PMC7738706

[ref55] Yang J. Wang C. Huang K. Zhang M. Wang J. Pan X. (2020). Compound *Lactobacillus* Sp. administration ameliorates stress and body growth through gut microbiota optimization on weaning piglets. Appl. Microbiol. Biotechnol. 104, 6749–6765. doi: 10.1007/s00253-020-10727-4, PMID: 32556411

[ref56] Yoon J. H. Ingale S. L. Kim J. S. Kim K. H. Lohakare J. Park Y. K.. (2013). Effects of dietary supplementation with antimicrobial peptide-P5 on growth performance, apparent total tract digestibility, faecal and intestinal microflora and intestinal morphology of weanling pigs. J. Sci. Food Agric. 93, 587–592. doi: 10.1002/jsfa.5840, PMID: 22903784

[ref57] Zhai Z. Ni X. Jin C. Ren W. Li J. Deng J.. (2018). Cecropin a modulates tight junction-related protein expression and enhances the barrier function of porcine intestinal epithelial cells by suppressing the MEK/ERK pathway. Int. J. Mol. Sci. 19:1941. doi: 10.3390/ijms19071941, PMID: 30004434 PMC6073479

[ref58] Zhai Z. Zhang F. Cao R. Ni X. Xin Z. Deng J.. (2019). Cecropin a alleviates inflammation through modulating the gut microbiota of C57bl/6 mice with DSS-induced IBD. Front. Microbiol. 10:1595. doi: 10.3389/fmicb.2019.01595, PMID: 31354682 PMC6635700

[ref59] Zhang L. Liu S. Piao X. (2021). Dietary 25-hydroxycholecalciferol supplementation improves performance, immunity, antioxidant status, intestinal morphology, and bone quality in weaned piglets. J. Sci. Food Agric. 101, 2592–2600. doi: 10.1002/jsfa.10889, PMID: 33063320

[ref60] Zhang M. Shan Y. Gao H. Wang B. Liu X. Dong Y.. (2018). Expression of a recombinant hybrid antimicrobial peptide magainin II-cecropin B in the mycelium of the medicinal fungus *Cordyceps militaris* and its validation in mice. Microb. Cell Fact. 17:18. doi: 10.1186/s12934-018-0865-3, PMID: 29402269 PMC5798188

